# Investigating the Mars–van Krevelen Mechanism for CO Capture on the Surface of Carbides

**DOI:** 10.3390/molecules30173637

**Published:** 2025-09-06

**Authors:** Naveed Ashraf, Younes Abghoui

**Affiliations:** Science Institute of the University of Iceland, 102 Reykjavik, Iceland

**Keywords:** carbides, electrocatalysis, CO reduction, DFT calculations, Mars–van Krevelen mechanism

## Abstract

Electrochemical reduction processes enable the CO to be converted into a useful chemical fuel. Our study employs density functional theory calculations to analyze the (110) facets of the transition metal carbide surfaces for CO capture, incorporating the Mars–van Krevelen (MvK) mechanism. All the possible adsorption sites on the surface, including carbon, metal, and bridge sites, were fully investigated. The findings indicate that the carbon site is more active relative to the other adsorption sites examined. The CO hydrogenation paths have been comprehensively investigated on all the surfaces, and the free energy diagrams have been constructed towards the product. The results conclude that the TiC is the most promising candidate for the formation of methane, exhibiting an onset potential of −0.44 V. The predicted onset potential for CrC, MoC, NbC, VC, WC, ZrC, and HfC are −0.86, −0.61, −0.61, −0.93, −0.87, −0.61, and −0.81 V, respectively. Our calculated results demonstrate that MvK is selectively relevant to methane synthesis. Additionally, we investigated the stability of these surfaces against decomposition and conversion to pure metals concerning thermodynamics and kinetics. It was found that these carbides could remain stable under ambient conditions. The exergonic adsorption of hydrogen on carbon sites, requiring smaller potential values for product formation, and stability against decomposition indicate that these surfaces are highly suitable for CO reduction reactions using the MvK mechanism.

## 1. Introduction

The increasing quantity of carbon dioxide (CO_2_) in the atmosphere poses a significant threat to human existence and global ecosystems. Despite the ongoing efforts of researchers and scientists, the current pace of progress remains insufficient, highlighting the urgent need for effective measures to mitigate this climate crisis. Human activities, specifically the combustion of fossil fuels, industrial processes, and vehicular emissions, are the primary sources of CO_2_ emissions. With the expansion of the world population, the need for energy concurrently increases [1]. To address this issue, there is an immediate need to reduce CO_2_ formation and transform it into valuable energy sources. CO_2_ reduction via the electrochemical method offers a promising route to transform CO_2_ into useful fuels and chemicals, thereby contributing to the closure of the global carbon loop. However, the electrochemical reduction of CO_2_ presents significant scientific challenges. One major hurdle is the difficulty in steering the reaction toward a single desired product, as multiple reduction pathways can occur simultaneously. In aqueous systems, the hydrogen evolution reaction (HER) often competes with the CO_2_ reduction reaction (CO_2_RR), reducing the overall selectivity of the process [2]. To overcome these limitations, the development of efficient and selective catalysts is crucial. Among various candidates, transition metal carbides (TMCs) are considered cost-effective and promising materials for heterogeneous catalysis [3]. These materials are valued for their remarkable chemical, thermal, and electrical properties, making them suitable for use in electrochemical CO_2_ conversion technologies [4].

These catalysts exhibit exceptional chemical, thermal, and structural stability, together with outstanding electrical and thermal conductivity [3,5,6]. TMCs display complex morphologies defined by an altered, adjusted electrical conductivity owing to the robust interplay between the carbon and metal atoms. As a result, these materials display unique properties concerning their transition metals and exhibit catalytic performance [7,8,9,10,11,12]. These TMC materials have transition metal, ionic crystal, and covalent solid properties [13] that can furnish catalytic characteristics for many applications, including the electrochemical formation of CO from CO_2_ and other compounds [14], hydrogen production [15], biogas formation [16], and benzene formation [17]. Previous research indicated that TMC prompted interest in several types of applications, such as desulfurization [18], dry reforming of methane [19], formation of methane into gas [12,20,21], and oxidation of CO [22]. The features of TMCs demonstrate potential for catalytic activities [23,24]. The conversion of CO_2_ into CO requires a 2-electron proton step, which can be easily achieved, but converting CO to a further useful product requires several electron-proton steps and high potentials. The primary objective of this work is to clarify the reaction pathway for the transformation of carbon monoxide (CO) into methane (CH_4_) via the MvK mechanism. This study aims to elucidate the thermodynamics of the CORR, free energy landscapes, and active surface sites that regulate CO hydrogenation to methane.

Prior studies suggest that TMC provides a substantial redox environment conducive to the reduction of CO into useful compounds, highlighting the capability of these catalysts [25,26]. This study is the inaugural comprehensive analysis of (110) facets of TMC to evaluate the phenomenon of CORR through the MvK mechanism. These carbides are typically in polycrystalline shapes, with the (100) and (110) facets being the predominant orientations. We have already examined the (100) facets, and here we concentrate on the (110) facets.

The concept of the MvK mechanism, originally proposed to describe oxidation catalysis over metal oxides, is characterized by the participation of lattice oxygen as the active oxidizing species. The oxidation of a substrate in this redox process results in the elimination of lattice oxygen, creating transitory oxygen vacancies on the catalyst surface. These vacancies enable the binding and activation of a gas-phase oxidant, usually molecular oxygen, which then replenishes the oxygen vacancies, thus reinstating the original oxidation state of the metal oxides and concluding the catalytic process.

Recent advancements have considerably expanded the MvK mechanism’s application beyond metal oxides, illustrating its relevance to many non-oxide systems, including metal sulfides, chlorides, nitrides, carbides, and hydrides. Specifically, metal carbides such as molybdenum carbide and iron carbide demonstrate MvK-type behavior in significant syngas-related transformations [27]. In chemical cyclic procedures, the homologation of methane, its transformation into other hydrocarbons, has been successfully performed by a two-step redox approach that includes metal carbide formation followed by reduction. Methane initially functions as the carbon supply and reducing agent, interacting with transition metals to produce metal carbides. Once created, these carbides undergo reduction with hydrogen, which promotes the liberation of hydrocarbon compounds [28]. A recently published study offers an in-depth look at the generation and regulations of surface vacancies in several catalytic materials, including oxides, carbides, nitrides, sulfides, and hydrides, relevant to the MvK mechanism [29,30,31]. The formation of intermediates, such as carbonates and hydroxyls, has been observed to participate in Mars–van Krevelen mechanisms. In the previous studies, the nitrogen reduction reaction has also been studied through the MvK mechanism theoretically [32,33,34,35] and experimentally for the nitrides [36], and this mechanism has been experimentally proven for Co_3_Mo_3_N catalysts [37]. These examples highlight the relevance of the Mars–van Krevelen (MvK) mechanism as a viable pathway in various catalytic reactions, particularly those involving transition metal carbides (TMCs), which are the focus of this study. This research aims to explore the stepwise protonation of the (110) surfaces of TMCs leading to methane formation, following the generation of surface carbon vacancies. The inherent carbon atoms in the carbide structure play a crucial role in facilitating methane evolution through interaction with four protons, which results in the creation of a carbon vacancy [38]. This vacancy is subsequently filled by the carbon atom from an adsorbed CO molecule, while the oxygen atom combines with protons to form water. This process effectively regenerates and cleans the catalytic surface, enabling continuous operation.

## 2. Methodology

Density functional theory (DFT) calculations were performed using version 6.3.2 of the Vienna Ab initio Simulation Package (VASP) [39], employing the Revised Perdew–Burke–Ernzerhof (RPBE) functional. The (110) surface orientation of transition metal carbides (TMCs) in the rocksalt (RS) crystal structure was selected for modeling. The RPBE functional offers a well-balanced trade-off between computational cost and accuracy, making it suitable for simulating surface reaction phenomena. Its reliability has been validated in prior studies focused on CO_2_RR, where predicted trends were found consistent with experimental observations [40,41,42].

**Figure 1:**
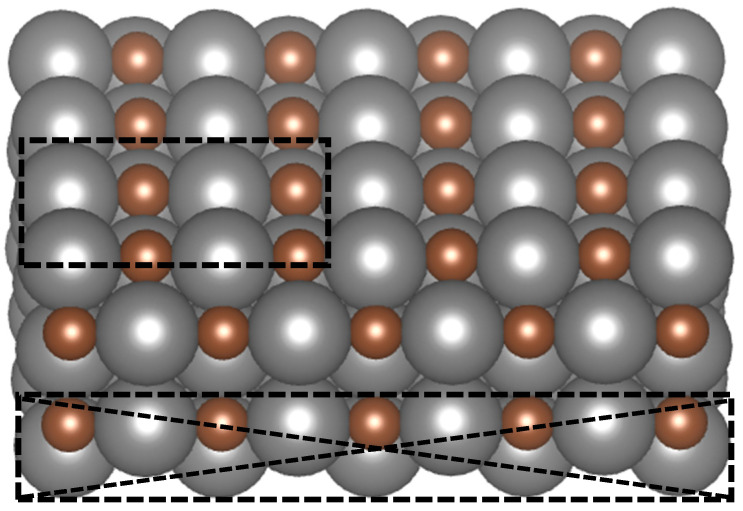
Illustration of the transition metal carbide (TMC) surface with a (110) orientation. Carbon atoms are represented in brown, while metal atoms are shown in gray. The dotted box at the top highlights the unit cell used for the study. The cross-hatched dotted region indicates that the bottom two layers were constrained during structural relaxation.

A 4 × 4 × 1 Monkhorst–Pack k-point was used for Brillouin zone sampling, with the cutoff energy of 400 eV. The surface slab consisted of five atomic layers and a total of 40 atoms, equally divided between metal and carbon atoms. Each layer contained four metal and four carbon atoms. A vacuum gap of 20 Å was introduced along the z-direction to avoid periodic interactions, while the x and y boundaries were kept periodic. As indicated in Figure 1, the upper three layers, along with adsorbates, were permitted to undergo complete relaxation during structural optimization, whereas the bottom two layers were constrained.

Reaction mechanisms for the CO reduction reaction (CORR) on TMC surfaces were explored using a thermochemical model (TCM), a well-established approach in the literature. The free energy diagrams for surface hydrogenation via the MvK mechanism along various pathways were computed with respect to the computational hydrogen electrode (CHE) introduced by Nørskov et al. [43]. The influence of applied electrochemical potential was incorporated through the (CHE) method. The Climbing Image Nudged Elastic Band (CI-NEB) method was used to examine carbon diffusion from the subsurface to the surface [44]. All catalyst structures were optimized until the forces converged below 0.03 eV/Å.

The formula employed to compute the adsorption energy of various species on the TMC surface is as follows [44]:

ΔE_ads_ = E_AB_ − E_A_ − E_B_ (1)

E_AB_ represents the total energy of a system with fully adsorbed intermediates and species on carbides, E_A_ defines the energy of the carbide catalyst in the absence of adsorbate, and E_B_ indicates the total energy of the adsorbate. To assess the thermodynamic feasibility of each reaction step, we calculated Gibbs free energy changes using standard DFT-based thermochemistry corrections, including zero-point energy, entropy, and pH adjustments.

ΔG = ∆E_DFT_ + ∆E_ZPE_ − T∆S + ∆G_pH_ + ΔH_0K→T_ (2)

∆E_DFT_ denotes the overall energy of the system as computed by DFT calculation, ∆E_ZPE_ signifies the calculated zero-point energy of the adsorbates. T∆S indicates the entropy shift between the beginning and final structures of each reaction. ∆G_pH_ denotes the energy correction of the H^+^ and is calculated as follows:

∆G_pH_ = ln10 × k_B_T × pH (3)

The ΔH_0K→T_ term denotes changes in the internal energy of the system due to the effect of temperature, which can be calculated by this formula $\int_{0}^{T}C_{p}\;(T^{\prime }\;)\;dT^{\prime }\;$, where $C_{p}\;(T^{\prime }\;)$ is the quantity of heat at constant pressure, and the integral of this equation suggests that at ambient temperature, the heat contribution is minimal. The values of the gas phase of the molecules were taken from the tables in the textbooks [45]. In our previous study, we already calculated the electronic properties of carbides [32]. The theoretical onset potential (OP) required to drive the reaction was calculated using the computational hydrogen electrode model by using Equation (4). The step with the highest free energy change (ΔG_max_) determines the limiting potential.

OP = −ΔG_max_/e (4)

## 3. Results and Discussion

### 3.1. Adsorption and MvK Mechanism

In the initial step of the study, all reactant species were adsorbed onto the pristine surfaces of the selected catalysts, evaluating adsorption at various potential active sites, namely the metal, bridge, and carbon sites across transition metal carbides (TMCs) such as CrC, MoC, NbC, VC, TiC, ZrC, HfC, and WC with the (110) surface orientation. The findings indicate that the carbon sites of the carbides show the highest catalytic progress, indicating more favorable exergonic adsorption energies as compared to the bridge and metal sites of the carbides. During the electrochemical process, surface poisoning may affect electrochemical performance, so investigation of the likelihood of O, H, and OH species binding the active sites on the surface is important. It was identified that with further protonation, O and OH convert into H_2_O_(l)_ via the following equations:

*O + H^+^ + e^−^ → *OH (5)

*OH + H^+^ + e^−^ → H_2_O_(l)_ (6)

**Figure 2:**
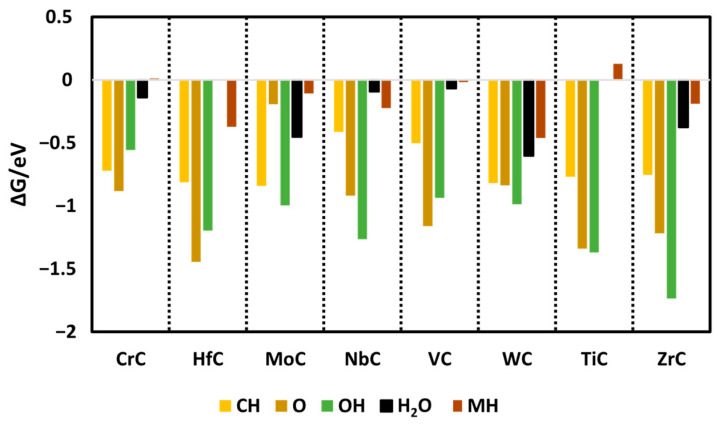
Calculated Gibbs free energies of all species on the surface of carbides. In the case of HfC and TiC, water does not bind.

This means that if O and OH adsorb on the surface of the carbides, it is of no concern, as further protonation steps convert them into H_2_O, which has weak adsorption on the surface as compared to other key species (shown in Figure 2). Hence, the likelihood of O and OH becoming poisonous is low.

**Figure 3:**
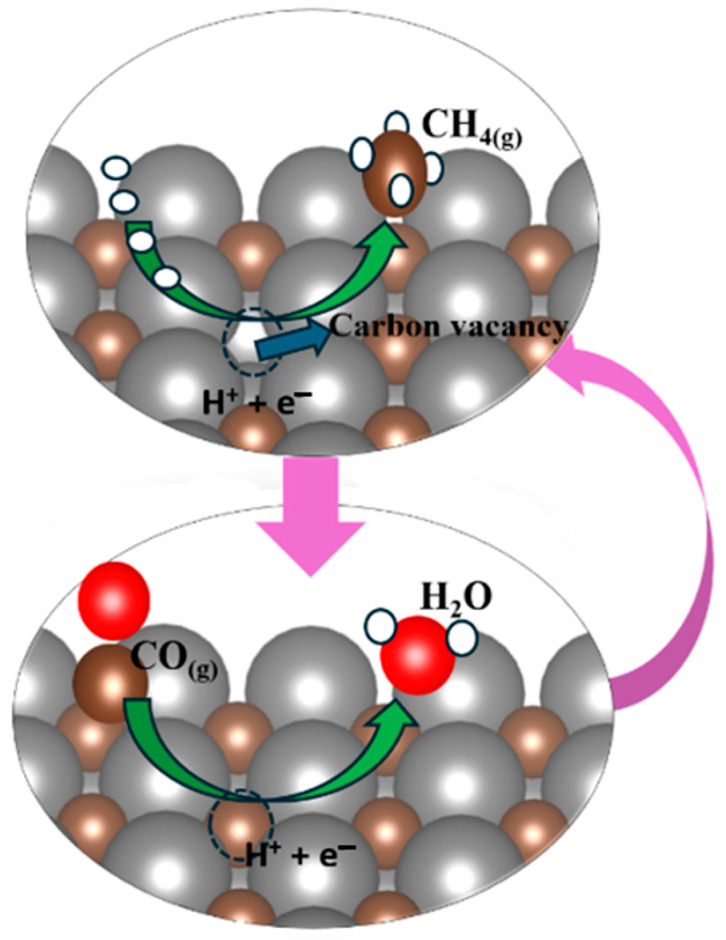
Representation of the generation of carbon vacancies via the MvK mechanism. The vacancy accommodates the C of atmospheric CO to fill the vacancy, and the O of CO is released in the form of water, and as a result, the surface will be regenerated and clean.

During the experimental synthesis of TMC, there is a possibility that a defect in the form of carbon can be formed, or there is a possibility that, during the electrochemical process, due to the continuous protonation, four protons attach to the C of carbides and release methane by leaving the carbon vacancy. Thus, the release of methane generates the carbon vacancy on the carbide’s surface. This carbon vacancy of carbides’ surface can be occupied by carbon from ambient CO, and one oxygen atom of CO is converted into a water molecule after finishing the electrochemical step with a clean and non-defective surface. This ongoing electrochemical cyclic process generates a product like methane, as illustrated in Figure 3.

The reactions associated with the MvK mechanism for the formation of the vacancy are specified in Equations (7) and (8), accompanied by the Kröger–Vink notation [46].

* + 4(H^+^ + e^−^) → CH_4(g)_ + ^C^Vac (7)

^C^Vac + CO + 2(H^+^ + e^−^) → H_2_O + * (8)

Here, the asterisk (*) represents the clean surface of the carbide catalysts, while “^C^Vac” denotes the carbon vacancy formed following methane (CH_4_) desorption.

### 3.2. Electrochemical Performance

**Figure 4:**
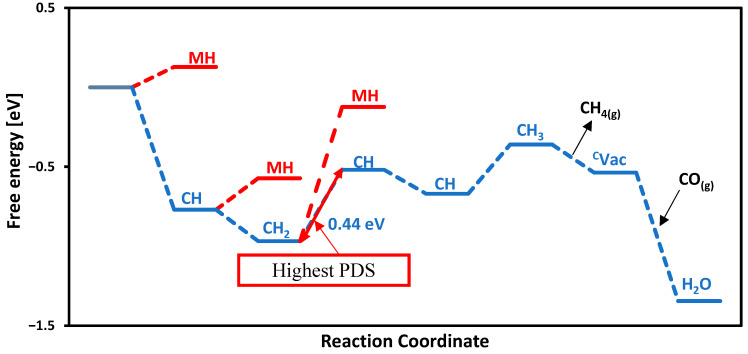
Calculated free energy diagram for CH_4_ production on the TiC surface via the MvK mechanism. CH is the proton on the carbon site, and MH is the proton on the metal site of TiC. The highest PDS is between CH_2_ and CH.

Among the TMC catalysts investigated, TiC stands out as the most suitable catalyst for CH_4_ production, demonstrating the least OP value of −0.44 V. Both CO adsorption and water formation proceed spontaneously, as depicted in the free energy profile presented in Figure 4. The initial protonation step occurs at the carbon site of TiC, leading to the exergonic formation of a CH intermediate with a free energy of −0.76 eV, thereby promoting CH_4_ generation. The most energy-demanding step, called the potential-determining step (PDS), is observed among the CH and CH_2_ intermediates (second carbon on the TiC surface), with an associated energy barrier of 0.44 eV.

**Figure 5:**
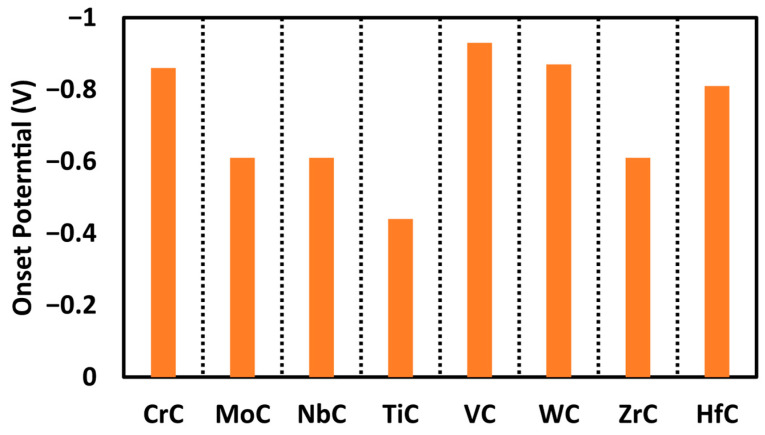
Comparison of the obtained onset potential for each catalyst for the production of methane.

In this investigation, we also evaluated the adsorption free energies of hydrogen atoms on various sites across the carbide surfaces. The results show that the proton adsorption on the metal site of TiC is endothermic with an adsorption free energy of 0.12 eV, as illustrated in the free energy profile in Figure 4 with the red path. While the adsorption of free energy of hydrogen was computed at each step, the carbon site consistently emerged as the most energetically favorable. We categorize the performance of materials on the basis of electrochemical performance, and our studied carbides are expected to produce methane at onset potentials below −1.0 V. The smaller (closer to zero) the onset potential, the better for CO reduction. The order of activity for the carbides studied here is as follows: TiC (−0.44 V) > MoC, NbC, and ZrC (−0.61 V) > CrC (−0.86 V) > WC (−0.87 V) > VC (−0.93 V), as shown in Figure 5. All of these surfaces are promising for CORR with onset potential below −1.0 V; however, TiC is the most promising surface not only due to the smaller onset potential needed but also due to the minimum number of proton-electron transfer steps to make methane.

**Figure 6:**
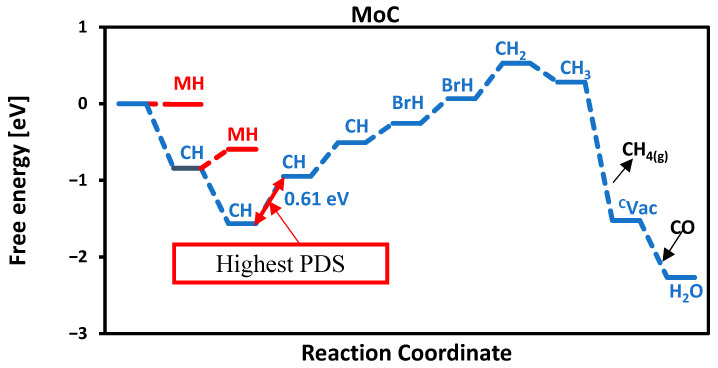
Free energy diagram of MoC. The highest PDS is between CH and CH.

At each protonation stage, all adsorption sites of carbides, such as carbon, metal, and bridge, were systematically explored. However, only the most thermodynamically favorable reaction pathways are reported. The MvK mechanism involves four proton–electron transfer steps to release methane. Depending on the specific catalyst, additional proton adsorption may occur on metal (MH), bridge (BrH), or different carbon sites (CH), as illustrated for MoC in Figure 6. In the first protonation step, a proton adsorbs on the first carbon atom. In the second step, a proton adsorbs on the second carbon atom, and this continues until all 4 carbon atoms of the surface are occupied by protons. In the fifth protonation step, the proton absorbs on the first bridge site, followed by adsorption on the second bridge site in the sixth step. The seventh protonation is more exergonic, as the first carbon forms a CH_2_ intermediate, which subsequently leads to the formation of CH_4_, as shown in Figure 6. For most surfaces studied, methane generation followed by carbon vacancy refilling via atmospheric CO is a facile process, effectively completing the catalytic cycle.

### 3.3. Vacancy Stability and Carbon Migration

**Figure 7:**
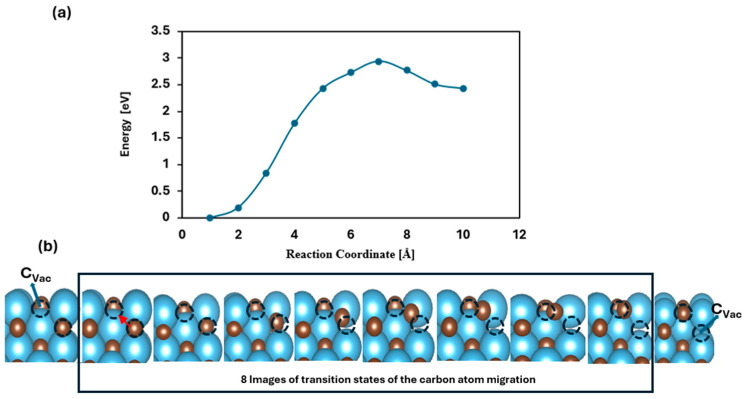
Schematic illustration depicting methane release and carbon migration: (**a**) shows the calculated energy barrier associated with the diffusion of a carbon atom from the subsurface layer to the top surface layer; (**b**) the first image illustrates the carbon vacancy (C_Vac_), the next eight images represent the transition states corresponding to the movement of carbon atoms of carbide from the 2nd layer toward the surface, and the last image represents the vacancy at the 2nd layer. The red arrow in the second image represents the direction of motion of the carbon atom from the sublayer towards the surface carbon vacancy.

Within the MvK framework, surface vacancies can extend into subsurface layers, potentially enabling the upward diffusion of the carbon atoms from the interior to occupy surface defects. If sustained, this process could eventually result in the depletion of bulk carbon atoms in the form of methane, effectively reducing the transition of metal carbide (TMC) to its pure metallic form. This study explores the thermodynamic feasibility and kinetic barriers associated with carbon migration from subsurface layers to the surface. To analyze the energy profile of this transformation, the CI-NEB method was performed to identify the minimum energy path and corresponding energy barriers [47]. Specifically, we calculated the activation energy (Ea) required for a carbon atom to migrate from the second to the topmost layer. Among the studied materials, TiC emerged as a noteworthy candidate, exhibiting a diffusion barrier of 2.94 eV. This high barrier indicates that carbon migration under ambient conditions is energetically demanding, confirming the structural robustness of TiC. Thus, both thermodynamic and kinetic considerations suggest that TiC is resistant to decomposition, as shown in Figure 7.

The energy difference between vacancies located at the surface and in the sublayer was determined using the equation ΔE = E_vac,2_ − E_vac,1_. The term E_vac,2_ corresponds to the vacancy energy of the sublayer of the carbides, and E_vac,1_ represents the vacancy energy on the surface. The results, summarized in Table 1, indicate that the catalysts demonstrate both thermodynamic and kinetic stability. Although the thermodynamic value for WC suggests it is thermodynamically unstable, further kinetic study shows that it is stable, as shown in Table 1. All of the kinetic values for the migration of carbon from the sublayer to the first layer in our studies are greater than 1.0 eV, which indicates that the rate of the migration reaction will be slow, and this indicates more stability.

### 3.4. Electronic Structure Analysis

**Figure 8:**
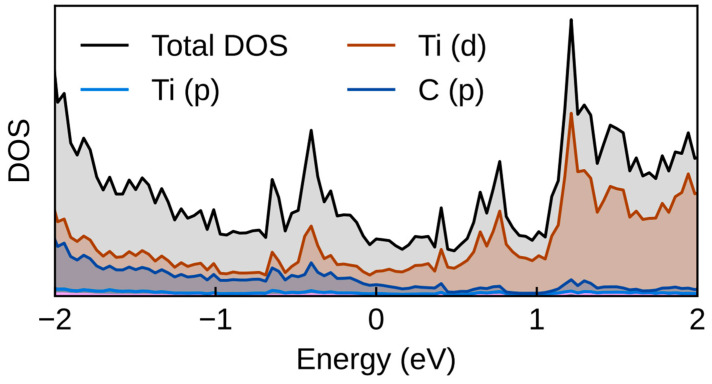
Calculated DOS of TiC.

Furthermore, we investigated the electronic properties of the materials by calculating the density of states (DOS) and performing charge analysis. Figure 8 illustrates the DOS for TiC, identified as the most promising catalyst. The results reveal an overlap between the carbon p orbitals and metal d orbitals, with states extending across the Fermi level—indicating the metallic nature of these transition metal carbides.

The computed Bader charges for hydrogen adsorbed on each carbide are as follows: VC (0.10), CrC (0.08), MoC (0.08), NbC (0.05), WC (0.02), HfC (0.02), TiC (0.01), and ZrC (0.02). A positive Bader charge reflects electron transfer from the catalyst surface to the adsorbed species, while a negative value indicates electron donation from the gas molecule to the surface. To calculate the charge density of H adsorption on our most promising candidate, TiC, we used Equation (9) [48].

**Figure 9:**
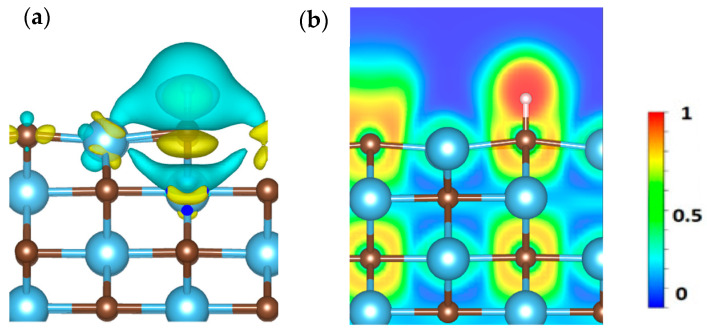
(**a**) represents the calculated charge density difference, and (**b**) represents the electron localization function (ELF) of H adsorption on the surface of the TiC.

Δρ = ρ_AB_ − ρ_A_ − ρ_B_ (9)

where Δρ indicates the total change in charge density, ρ_AB_ represents the charge density of adsorbed H on the surface of TiC, ρ_A_ is the charge density of TiC, and ρ_B_ is the charge density of H. Yellow and cyan colors are used to show charge accumulation and depletion during H adsorption on TiC as depicted in Figure 9a. Furthermore, the electron localization function (ELF) has been computed to assess the electron density behavior of hydrogen on the TiC surface, as illustrated in Figure 9b, which facilitates the differentiation between confined regions and more dispersed areas. The ELF varies from 0 to 1, indicating total delocalization (as observed in metallic systems) as well as strong localization (as seen in covalent interactions) [49]. Our findings elucidate the collective electron interactions, emphasizing the attributes of covalent bonding.

### 3.5. Experimental Validation

In the previous studies, theoretical works demonstrated how DFT calculations are helpful to provide key guidelines to experimentalists. For example, in a previous study, Ti_2_C_3_ and Mo_2_C were investigated theoretically and experimentally for CO_2_ reduction. The DFT calculations indicated that intermediates were chemisorbed on the surface of catalysts, which is challenging for reduction towards further intermediates and products [50]. In addition, experimental studies for Ti_2_CT_x_ (T_x_ = F and O) and Mo_2_CT_x_ were carried out for formic acid formation at −1.0 V, and then computational studies via DFT calculations explained how the termination group (-F) helped to tune the adsorption strength of CO_2_ and its intermediates [51]. This study also gives a detailed analysis for experimenters on how carbon on the surface of the carbides causes the formation of CH_4,_ and the carbon vacancy accommodates the atmospheric CO to fill the vacancy to regenerate the surface.

## 4. Conclusions

Transition metal carbides (TMCs) with (110) facets have been widely explored as catalysts for carbon monoxide reduction reactions. To identify the most reactive surface sites, we calculated the adsorption free energies of various species on all potential adsorption sites, including carbon, bridge, and metal positions. Our findings reveal that carbon sites exhibit superior activity compared to other examined locations. To further evaluate catalytic performance, we investigated the MvK mechanism for CO reduction on TMC surfaces. The results highlight the relevance of the MvK mechanism for methane production, with TiC emerging as a particularly effective catalyst, showing an onset potential of −0.44 V. The onset potential was determined to be −0.93 V for VC, −0.86 V for CrC, −0.87 V for WC, −0.81 V for HfC, and −0.61 V for MoC, NbC, and ZrC, respectively. Additionally, we evaluated the structural stability of these carbides and the persistence of surface vacancies by analyzing both the thermodynamic and kinetic aspects of carbon atom migration from sublayers to the surface. The calculated diffusion barriers indicate that this migration is energetically demanding, confirming the robust stability of these catalysts. The favorable hydrogen adsorption on carbon sites, low onset potentials, and substantial diffusion barriers collectively suggest that TMC (110) surfaces are highly suitable for methane production via the MvK pathway.

## Figures and Tables

**Table 1:** Calculated thermodynamics and kinetics values of migration of a carbon atom from the sublayer to the first layer.

Catalyst	CrC	HfC	MoC	NbC	TiC	VC	WC	ZrC
E_a_	2.77	3.41	2.21	2.46	2.94	1.46	1.92	3.28
ΔE	1.37	2.62	0.1	1.35	2.43	1.37	−0.68	2.62

## Data Availability

The original contributions presented in this study are included in the article. Further inquiries can be directed to the corresponding authors.
